# Should Patients Be Confident in Their Efficacy in Improving Their Functional Abilities After a Stroke?

**DOI:** 10.7759/cureus.51105

**Published:** 2023-12-26

**Authors:** Heltty Heltty, Cholik Harun Rosjidi, Lisnawati Lisnawati

**Affiliations:** 1 Medical Surgical Nursing, Universitas Mandala Waluya, Kendari, IDN; 2 Medical Surgical Nursing, Sekolah Tinggi Ilmu Kesehatan Karya Kesehatan, Kendari, IDN; 3 Community Nursing, Universitas Mandala Waluya, Kendari, IDN

**Keywords:** self-efficacy, cross-sectional studies, confident, functional ability, stroke

## Abstract

Background

Patients’ self-confidence in their abilities needs to be improved to achieve functional abilities after a stroke. Self-efficacy is a prerequisite for self-management after a stroke. This study aimed to analyze the relationship between self-efficacy and the functional abilities of post-stroke patients.

Methodology

This was an analytical cross-sectional study conducted over two months. A total of 145 respondents were recruited using the convenience sampling method. Respondents were post-stroke patients who had undergone the post-stroke phase during the first three to six months since the acute stroke. Data collection was performed through questionnaire interviews. Data were analyzed using descriptive analysis and Spearman correlation.

Results

There was a significant positive relationship between self-efficacy and functional independence (p < 0.05).

Conclusions

Self-efficacy influences motivation to perform activities of daily living, which can increase the achievement of functional abilities.

## Introduction

Achieving independent functional abilities after stroke is important in the care of post-stroke patients. Achieving functional independence can be seen in the patient’s ability to perform daily activities. Basic activities of daily living (ADLs) function as an important prognostic factor of functional independence during early post-stroke recovery [1]. The inability of post-stroke patients to perform physical activities can result in death. The death of post-stroke patients is closely related to physical dependence, lack of activity, worsening of the disease, and disturbances in body balance [2]. The results of a meta-analysis showed that post-stroke patients who performed physical activity have a 27% lower risk of recurrent stroke and death compared to individuals who were not active [3]. Physical activity can improve cardiovascular capacity, mobility, balance, walking ability, muscle strength, and overall quality of life, thereby reducing disability and the risk of recurrent stroke [4].

Longitudinal studies showed that most functional recovery occurs within the first month after stroke, but recovery declines between three and six months post-stroke [5,6]. Another study reported that post-stroke patients who were independent in the first three months could become dependent within one year if they did not do physical activity with a decline rate of 3% per year [7]. Therefore, it is necessary to achieve functional independence. Efforts to achieve functional independence are part of self-management. Post-stroke patients require ongoing self-management to restore lost functional abilities. Applying self-management principles in inpatient rehabilitation can support the development of internally motivated thought patterns and behavior to increase patient engagement [8]. Previous research has found that involving patients in their care is significantly related to achieving functional independence [9]. However, to improve the self-management abilities of post-stroke patients, self-efficacy is needed. Self-efficacy is a prerequisite for the success of effective self-management [10].

Self-efficacy relates to “belief in one’s ability to organize and carry out the actions necessary to produce certain achievements” [1]. Studies on chemotherapy patients found that better self-efficacy was associated with better quality of life and lower fatigue [11]. Confidence in one’s ability to manage oneself well is an important aspect of ongoing care [12]. Individuals with high self-efficacy after a stroke usually have greater confidence to participate in ADLs, a higher ability to overcome obstacles in their recovery, and usually have better psychosocial functioning and well-being compared to those with low self-efficacy [1]. Self-efficacy increases cancer patients’ confidence in performing self-care behavior during chemotherapy [11]. For this reason, this study aims to analyze the relationship between self-efficacy and patients’ functional abilities in the first three to six months after a stroke.

## Materials and methods

Study design, sample, and setting

This analytical cross-sectional study was conducted over two months. Patient data were obtained from Bahteramas Hospital. A total of 145 respondents were recruited using convenience sampling methods. This method was carried out based on the accessibility and availability of researchers, where the sample was recruited from the hospital that had the largest number of stroke patients and the location of the respondents’ homes where they could be reached by researchers. Inclusion criteria were patients aged 25-65 years, who had experienced the post-stroke phase during the first three to six months since the acute ischemic stroke, did not experience cognitive impairment, were willing to participate in the research, were in a stable condition, did not experience post-stroke depression as proven by measurements using the Patient Health Questionnaire-9 (PHQ-9), and lived with their family (partner and/or children) who could act as caregivers. Patients with severe weakness and aphasia were excluded from this study. Respondents were recruited at the Bahteramas Hospital Rehabilitation Unit and then assessed at home to measure their functional abilities. Researchers explained the objectives and rights of respondents during the study, including explanations relating to confidentiality, anonymity, and confidentiality of their personal data.

Study instrument

The instruments used in this research were the Stroke Self-Efficacy Questionnaire (SSEQ) and the Modified Barthel Index (MBI). The SSEQ is used to measure respondents’ self-efficacy. The SSEQ contains 13 questions that assess self-efficacy beliefs related to daily tasks and self-management (e.g., mobility, ambulation, dressing, coping with stroke frustration, and exercise adherence). The SSEQ has high internal consistency (Cronbach’s alpha = 0.90) [1,13]. It uses a 10-point scale, where 0 implies not confident at all to 10 implies very confident [14].

The MBI is used to measure behavior related to daily living activities and includes 10 items, namely, feeding, grooming, bathing, dressing, toilet transfer, bladder control, bowel control, chair/bed transfer, stair climbing, and ambulation. It is recommended for clinical and research applications because it has better test-retest reliability and relatively lower random measurement error than the native Barthel Index (BI) [15]. The MBI is also recommended for clinical and research use as an outcome measure for stroke patients [16]. It has better sensitivity and reliability (Cronbach’s alpha = 0.93) [17]. The total MBI assessment score ranges from 0 to 100. A lower score indicates less independence, whereas a higher score indicates greater independence [18]. A maximum score of 100 indicates the patient is completely independent in performing basic ADLs, whereas a score of 0 indicates total dependence [18]. The MBI score is divided into five categories: 0-20 (total dependence), 21-60 (severe dependence), 61-90 (moderate dependence), 91-99 (slight dependence), and 100 (independence) [19].

Data collection

Data collection was performed from May to June 2023 through visits to respondents’ homes. Data on self-efficacy and functional abilities of respondents were studied through questionnaire interviews. The researcher explained each question item to the respondent. Validation of the respondent’s functional ability data was also done by asking the respondent’s family or caregiver who lived at home with them about their functional ability achievements.

Data analysis

Demographic data, clinical information, self-efficacy, and functional ability achievement were analyzed using descriptive analysis. The relationship between self-efficacy and functional ability was analyzed using Spearman correlation. All data were analyzed using SPSS Statistics version 25 (IBM Corp., Armonk, NY, USA) at a significance level of p < 0.05.

Ethical considerations

This study was approved by the ethics committee of Bahteramas Hospital (IRB1460-04). All respondents signed a consent form to participate in the research after receiving an explanation about the study.

## Results

The majority of the respondents were over 50 years old (57.2%) and female (53.8%), high school graduates (44.8%), married (58.6%), experienced hemiparesis on the left side of the body (60%), and right-handed (89.0%). All patients had weakness in at least one muscle group, mostly on the left side (left arm = 79.3%). The majority of the muscle strength groups displayed active movement against gravity and resistance (Medical Research Council grade = 4). The most common comorbidity was hypertension (41.4%). Based on the National Institutes of Health Stroke Scale scores, most respondents had moderate stroke severity (71%). Most respondents also had moderate dependence in performing ADLs (42.1%) and good self-efficacy (53.1%) (Table 1). Determining the self-efficacy category using a cut-off point, the median value was 84.0.

**Table 1:** Characteristics of respondents. DM = diabetes mellitus

Characteristics of respondents		N	%
Age (mean ± SD)		50.45 ± 12.263	
	50 years old or younger	62	42.8
	>50 years old	83	57.2
Gender	Women	78	53.8
	Man	67	46.2
Last education	College	47	32.4
	High school	65	44.8
	Middle/Elementary school	33	22.8
Marital status	Married	85	58.6
	Widow/Widower/Single	60	41.4
Type of hemiparesis	Hemiparesis on the left body side	87	60.0
	Hemiparesis on the right body side	58	40.0
Comorbidities	Hypertension	60	41.4
	Hypertension and DM	49	33.8
	Hypertension, DM, and hypercholesterolemia	36	24.8
Stroke severity	Minor stroke	42	29.0
	Moderate stroke	103	71.0
Handedness	Left-handed	16	11.0
	Right-handed	129	89.0
Functional independence	Severe dependence	54	37.2
	Moderate dependence	61	42.1
	Slight dependence	30	20.7
Self-efficacy	Low self-efficacy	68	46.9
	Good self-efficacy	77	53.1
Frequency of muscle weakness	Right arm	103	71.0
	Left arm	115	79.3
	Right leg	99	68.3
	Left leg	111	76.6
Muscle strength (median (range))	Right arm	4 (2–5)	
	Left arm	4 (2–5)	
	Right leg	4 (3–5)	
	Left leg	4 (3–5)	

Table 2 shows that self-efficacy had a significant relationship with the patient’s functional independence in performing daily activities (p = 0.000). The correlation test results showed a very strong relationship between self-efficacy and functional independence. This relationship was also positive, meaning that the higher the self-efficacy, the higher the respondent’s achievement of functional independence.

**Table 2:** Relationship between self-efficacy and functional independence.

Self-efficacy	Functional independence			Total (n = 145)	P-value	R
	Severe dependence (n = 54), n (%)	Moderate dependence (n = 61), n (%)	Slight dependence (n = 30), n (%)			
Low self-efficacy	51 (75.0)	17 (25.0)	0 (0)	68	0.000	0.957
Good self-efficacy	3 (3.9)	44 (57.1)	30 (39.0)	77		

Table 3 shows that among the 13 dimensions of respondents’ self-efficacy, the highest dimension was “Do your own exercise program every day” (mean = 6.31, SD = 1.357) and the lowest dimensions were “Prepare the food you want for yourself” and “Continue to get faster at tasks that have become slower since your stroke” (mean = 5.77). Regarding functional independence, the highest was “Chair/bed transfer ability” (mean = 9.84, SD = 4.231) and the lowest was “Bathing ability” (mean = 2.99, SD = 1.470).

**Table 3:** Descriptive statistics of self-efficacy and functional independence.

Variable	Average score range (minimum–maximum)	Mean	SD
Self-efficacy: Respondents confident			
Get comfortable in bed every night	4–8	6.28	1.552
Out of bed on your own even if you feel tired	4–8	6.21	1.542
Walk a few steps on any surface in your home by yourself	4–8	6.16	1.461
Walk around your house to do as many things as you want	4–8	6.08	1.479
Walk safely outside alone on any surface	4–8	5.92	1.431
Use both hands to eat your food	4–9	6.08	1.472
Dress and undress yourself even when you feel tired	4–8	5.93	1.489
Prepare the food you want for yourself	4–8	5.77	1.471
Persevere to make progress on your stroke after discharge from therapy	4–8	6.21	1.435
Do your own exercise program every day	4–8	6.31	1.357
Overcome the frustration of not being able to do some things because of your stroke	4–9	6.22	1.446
Continue doing most of the things you enjoyed before the stroke	3–9	6.15	1.639
Continue to get faster at tasks that have become slower since your stroke	3–9	5.77	1.472
Functional independence			
Chair/bed transfers ability	3–15	9.84	4.231
Ambulation ability	3–15	8.94	4.634
Stairs climbing ability	0–8	4.33	3.076
Toilet transfers ability	2–8	5.35	2.610
Bowel control ability	2–10	5.37	3.115
Bladder control ability	0–10	4.24	3.552
Bathing ability	1–5	2.99	1.470
Dressing ability	2–8	5.70	2.424
Personal hygiene ability	1–5	3.11	1.329
Feeding ability	2–8	5.89	2.211

## Discussion

Based on the results of this study, there was a significant relationship between self-efficacy and functional independence (p < 0.05). This is in line with other research that there is a relationship between functional independence and self-efficacy, where functional independence strongly predicts the quality of life of stroke sufferers and high self-efficacy determines the quality of life [20]. Stroke sufferers with a higher level of self-efficacy have the potential to recover gradually during the rehabilitation process [21]. Physical activity stimulates neuroplasticity and improves patient function [13].

In this study, 57.1% of good self-efficacy respondents experienced moderate dependence. Achieving this ability is closely related to the individual’s motivation to perform physical activity. Motivation is closely related to self-efficacy, which refers to an individual’s desire to perform certain behaviors to achieve their goals [1]. Motivation is a process where self-efficacy influences individual function and has an impact on post-stroke rehabilitation outcomes [1]. The dimension “do exercise program every day” is the dimension that is highest owned by respondents, which shows the high motivation that respondents have. A person’s level of self-efficacy influences their motivation to achieve their goals, determines how long the individual persists when facing challenges, and determines their resilience to failure [1]. Motivation can be provided by family or people closest to the patient. In this study, respondents lived with their families who could act as a support system for respondents so that they could increase their self-efficacy.

However, 42.1% of respondents experienced moderate dependence in this study. This percentage is higher than respondents who experienced severe dependence and slight dependence. Recovery of post-stroke activity limitations mostly occurs within a certain duration, especially the first 12 months after stroke [22]. Respondents in this study were in the first three to six months of recovery after a stroke. Although several studies state that slow recovery occurs in the first three to six months after a stroke, this study showed an increase in the respondents’ physical activity abilities. This can be related to the degree of stroke severity experienced by the respondent. Patients who experience mild strokes on average experience better recovery than stroke patients with severe deficits [23]. As many as 71.0% of respondents in this study experienced moderate stroke severity.

In the functional independence dimension, the ability “chair/bed transfer ability” was the highest achievement item (mean = 9.84) in this study. In performing ADLs for stroke patients, moving between bed and wheelchair is not as difficult as walking [24]. Functional recovery is relatively better when compared to motor recovery [25]. Transferability is included in functional recovery. Transfer is considered one of the skills acquired through motor learning [24]. Self-efficacy develops due to the learning process. Individual self-efficacy plays a major role in the approach to goals, tasks, and challenges [26]. This can have a positive impact on self-confidence and belief in one’s ability to do something. Self-efficacy is a mediator of the relationship between driving performance and activity participation after stroke, so the contribution of self-efficacy is crucial [21].

The findings from this study have several practical implications for improving patient self-efficacy. Providing information to patients and families can improve their ability to care for themselves and their families. In addition, it is necessary to provide positive reinforcement for the patient’s achievements so that they increase their confidence in their abilities. However, this study had several limitations, including the limited sample because the recruitment duration was only two months and the heterogeneity of hemiparesis experienced by respondents. This study only applies to ischemic stroke patients. Future research needs to add a larger sample size, the duration of sample recruitment needs to be increased to at least six months, and the homogeneity of stroke lesions needs to be considered.

## Conclusions

Self-efficacy contributes to improving the functional abilities of post-stroke patients. This shows improved recovery after a stroke. Self-efficacy is influenced by the motivation to perform certain behaviors, in this case, daily life activities. Performing daily living activities can increase the achievement of functional abilities. Achieving this functional ability cannot be separated from the patient’s involvement in self-care. In patient-centered care, as individuals, patients play an active role and as a resource that contributes to their care. Increasing the patient’s knowledge, skills, and awareness of their ability to provide care for themselves is crucial. Apart from that, there is a need for support from various parties (health workers, family, and peers) to increase self-confidence in patient self-efficacy.

## References

[1] Gangwani R, Cain A, Collins A, Cassidy JM (2022). Leveraging factors of self-efficacy and motivation to optimize stroke recovery. Front Neurol.

[2] Weng SC, Hsu CY, Shen CC, Huang JA, Chen PL, Lin SY (2022). Combined functional assessment for predicting clinical outcomes in stroke patients after post-acute care: a retrospective multi-center cohort in central Taiwan. Front Aging Neurosci.

[3] Hou L, Li M, Wang J (2021). Association between physical exercise and stroke recurrence among first-ever ischemic stroke survivors. Sci Rep.

[4] Gagnon MA, Batcho CS, Best KL (2022). A description of physical activity behaviors, barriers, and motivators in stroke survivors in Quebec. Disabil Health J.

[5] Heltty H, Zahalim Z (2023). Resilience after stroke and its correlation with functional independence. J Ners.

[6] Yun SM, Lee SY, Sohn MK (2020). Factors associated with changes in functional independence after six months of ischemic stroke. Brain Neurorehabil.

[7] Vieira A, Soares P, Nunes C (2021). Predicting independence 6 and 18 months after ischemic stroke considering differences in 12 countries: a secondary analysis of the IST-3 trial. Stroke Res Treat.

[8] Kenah K, Tavener M, Bernhardt J, Spratt NJ, Janssen H (2023). "Wasting time": a qualitative study of stroke survivors' experiences of boredom in non-therapy time during inpatient rehabilitation. Disabil Rehabil.

[9] Heltty H (2022). Patient, family, and peer engagement in nursing care as an effort to improve the functional independence of post-stroke urinary incontinence patients: a cross-sectional study. Cureus.

[10] Fan X, Xing C, Yang L, Wang J, Feng L (2020). Fatigue, self-efficacy and psychiatric symptoms influence the quality of life in patients with myasthenia gravis in Tianjin, China. J Clin Neurosci.

[11] Akin S, Kas Guner C (2019). Investigation of the relationship among fatigue, self-efficacy and quality of life during chemotherapy in patients with breast, lung or gastrointestinal cancer. Eur J Cancer Care (Engl).

[12] Kim S (2021). The effects of self-efficacy on cognitive function in patients with acute stroke: verification of the medicating effect of family support. Am J Nurs Sci.

[13] Błaszcz M, Prucnal N, Wrześniewski K (2022). Physical activity, psychological and functional outcomes in non-ambulatory stroke patients during rehabilitation-a pilot study. J Clin Med.

[14] Riazi A, Aspden T, Jones F (2014). Stroke Self-efficacy Questionnaire: a Rasch-refined measure of confidence post stroke. J Rehabil Med.

[15] Yang CM, Wang YC, Lee CH, Chen MH, Hsieh CL (2022). A comparison of test-retest reliability and random measurement error of the Barthel Index and modified Barthel Index in patients with chronic stroke. Disabil Rehabil.

[16] Wang YC, Chang PF, Chen YM, Lee YC, Huang SL, Chen MH, Hsieh CL (2023). Comparison of responsiveness of the Barthel Index and modified Barthel Index in patients with stroke. Disabil Rehabil.

[17] Küçükdeveci AA, Yavuzer G, Tennant A, Süldür N, Sonel B, Arasil T (2000). Adaptation of the modified Barthel Index for use in physical medicine and rehabilitation in Turkey. Scand J Rehabil Med.

[18] Lee SY, Kim DY, Sohn MK (2020). Determining the cut-off score for the Modified Barthel Index and the Modified Rankin Scale for assessment of functional independence and residual disability after stroke. PLoS One.

[19] Shah S, Vanclay F, Cooper B (1989). Improving the sensitivity of the Barthel Index for stroke rehabilitation. J Clin Epidemiol.

[20] Ogwumike OO, Omoregie AA, Dada OO, Badaru UM (2022). Quality of life of stroke survivors: a cross-sectional study of association with functional independence, self-reported fatigue and exercise self-efficacy. Chronic Illn.

[21] Honado AS, Atigossou OL, Roy JS, Daneault JF, Batcho CS (2023). Relationships between self-efficacy and post-stroke activity limitations, locomotor ability, physical activity, and community reintegration in Sub-Saharan Africa: a cross-sectional study. Int J Environ Res Public Health.

[22] Preston E, Ada L, Stanton R, Mahendran N, Dean CM (2021). Prediction of independent walking in people who are nonambulatory early after stroke: a systematic review. Stroke.

[23] Grefkes C, Fink GR (2020). Recovery from stroke: current concepts and future perspectives. Neurol Res Pract.

[24] Kitamura S, Otaka Y, Murayama Y (2022). Difficulty of the subtasks comprising bed-wheelchair transfer in patients with subacute strokes: a cohort study. J Stroke Cerebrovasc Dis.

[25] Verheyden G, Nieuwboer A, De Wit L (2008). Time course of trunk, arm, leg, and functional recovery after ischemic stroke. Neurorehabil Neural Repair.

[26] Ferari F (2023). Skills mismatch and change confidence: the impact of training on change recipients’ self-efficacy. Eur J Train Dev.

